# General Mapping of the Environmental Performance in Climate Change Mitigation of Spanish Universities through a Standardized Carbon Footprint Calculation Tool

**DOI:** 10.3390/ijerph191710964

**Published:** 2022-09-02

**Authors:** Antonio Guerrero-Lucendo, Fuensanta García-Orenes, Jose Navarro-Pedreño, David Alba-Hidalgo

**Affiliations:** 1 Environmental and Sustainable Development Area, Miguel Hernandez University of Elche, Avda. de la Universidad s/n, 03202 Elche, Spain; 2 Department of Agrochemistry and Environment, Miguel Hernandez University of Elche, Avda. de la Universidad s/n, 03202 Elche, Spain; 3 Specific Didactics Department, Faculty of Education, Autonomous University of Madrid, 28049 Madrid, Spain

**Keywords:** carbon footprint, sustainability, greenhouse gas, climate change, higher educational institutions, university, environmental performance, benchmarking

## Abstract

Higher education institutions (HEIs) can be considered role models of small cities that contribute to the fight against climate change. Therefore, assessing their own carbon footprints (CFs) and drawing conclusions gives significance to this study. In this study, 77 CFs from 14 HEIs were obtained through a tool developed by the Spanish Government. They were analyzed along with different variables and recalculated using the same standardized activity ratios. As a result, a general mapping of the environmental performance in climate change mitigation of Spanish universities has been obtained. Although there is an overall decrease in total CF (72.7%), direct greenhouse gas (GHG) emissions (Scope 1) remain stable, while the decrease is due to the reduction of emissions caused by electricity consumption (Scope 2) through the use electricity suppliers that guarantee the energy provided is generated from renewable sources. A lack of consensus in the definition of “student” and “employee”, used for the activity ratios, causes large variations in the relative CF values. For worldwide benchmarking of HEIs’ climate change performance, CF can be a valid indicator only if they: (1) include standardized Scope 1 and 2 emission sources, (2) use the same emission factors, and (3) calculate activity ratios from standardized functional units.

## 1. Introduction

Higher education institutions (HEI) or universities, which can be seen as small cities contributing to sustainability through their education, research, the operation of their own estate, and their relationship with society [1,2,3], play an important role in environmental sustainability through combating climate change [4]. However, they are beginning to address climate issues through carbon reduction policies [5].

Measuring sustainability is a complex and challenging process for HEIs worldwide [6]. Moreover, the lack of a global approach on what to measure is present in the results on the contribution to sustainability of Spanish universities [7]. Although the definition of environmental performance is manifold in the existing literature [8], some studies suggest that the ISO 14000 series provides an accepted definition that encompasses both management activities with respect to environmental aspects, and the results of these activities and processes [9,10,11]. Even so, there is little consensus on how to measure elements of environmental performance, being still far from standardized [8,12,13].

Environmental performance indicators must have certain characteristics and consider certain properties to ensure their usability, comparability, and consistency. The carbon footprint (CF) meets these characteristics if properly assessed and interpreted, and it is the key performance indicator commonly used to assess environmental impacts related to climate change [14].

The assessment of the calculation of CF in HEIs has been considered as one of the initial steps towards campus environmental sustainability [15,16], and CF reporting the start of sustainable educational practices [17]. The CF of an organization can be defined as the measurement of total greenhouse gas (GHG) emissions caused directly and indirectly due to its activity [18]. An activity can have several sources of GHG emissions, usually classified in three scopes: (1) direct emissions, (2) indirect emissions for electricity consumption, and (3) other indirect emissions [19]. It is expressed as the amount of CO_2_ equivalent (CO_2_eq), a universal unit of measurement that indicates the global warming potential (GWP) of each one of the different GHGs (CO_2_, CH_4_, N_2_O, HFCs, etc.).

Although there are no relevant references that evidence the use of CF as an environmental performance indicator in HEIs [14], the assessment of CF in these organizations is widely registered: The literature contains a few studies focused on comparing the CFs of different HEIs belonging to specific associations in a specific geographical area [16], such as Bailey and LaPoint [20], who compared the CF from nine universities located in Texas (USA) or Robinson et al. [21] with 20 colleges in the United Kingdom. In some cases, they are very detailed studies, but pertaining to a single university, such as Abdelalim et al. [22], which reported individual CFs for 42 buildings on the Carleton University campus in Canada. Globally, there are also few reviews of HEIs CFs, e.g., Helmers et al. [23] and Valls-Val and Bovea [24] who studied and compared with corrections and amendments the CFs of 20 and 35 universities worldwide, respectively. At the Spanish level, there are several publications of articles about university CF in recent years, such as for the Polytechnic University of Valencia [14], the School of Forestry Engineering of the Polytechnic University of Madrid [25], the University of Castilla-La Mancha [26], the University of the Basque Country [27], the Miguel Hernández University of Elche [28], and the Jaime I University [24]. Prior to these, the literature review provides broader studies focused on the calculation of the so-called “Ecological Footprint” [29,30,31,32], which provides indicators aggregated into a single index, where the result is biased by the criteria applied in the evaluation [33]. Although some CF publications make brief comparisons of their results with other universities, no comparative reviews of the CFs of Spanish HEIs in the last decade have been found.

In most of the cases universities use their own calculation methods and do not use specific tools, or if they use tools, there is no consensus on which one to use from the wide variety available [24], so comparisons with previous literature are limited [26]. Consequently, there appears a lack of standardization due to the use of different emission factors, as well as a lack of normalized results due to the selection of different functional units (students, employees, areas, budgets, etc.) [16]. It is recommended to continue researching in order to set emission factors, and the methodology would be standardized, and more accurate comparisons could be established between related studies [27].

To make a comparative evaluation of the operational environmental performance of HEIs in terms of climate change, a standardization of functional units is also required to normalize the results despite the size of the HEI [34], as well as in the choice of emission sources and emission factors [23]. In this regard, the Spanish Carbon Footprint Registry (SCFR), which was approved by the Spanish Government [35] through its Ministry of Agriculture, Food, and Environment, tries to eliminate this gap in standardization, as it is required that the emission factors of fossil fuels, the global warming potentials of the different gases of air conditioning systems, and the emission factor associated with each of the electricity companies are those officially provided by the Government. The SCFR aims to combat climate change by promoting the calculation and reduction (as it requires the existence of a reduction plan) of the CF of Spanish organizations in sectors not covered by the EU Emissions Trading Scheme (ETS), such as agriculture, waste management, or universities (Directive 2003/87/EC). Subsequently, the Government, through the Spanish Climate Change Office, verifies the results and issues a seal certifying their inclusion in the registry and the level of commitment achieved (calculation, reduction, and/or offsetting).

Considering the opportunity for standardization offered by the SCFR as a dual assessment tool for both management and operational performance, the aim of this study is to carry out a general mapping of the Spanish University System’s effort in climate change mitigation and to find a detailed proposal of activity ratios and emission sources specific to HEIs around the world, allowing for subsequent comparison of efforts and results. Furthermore, it will facilitate decision-making at universities to address their contribution to climate change based on this performance indicator.

## 2. Materials and Methods

**Figure 1:**
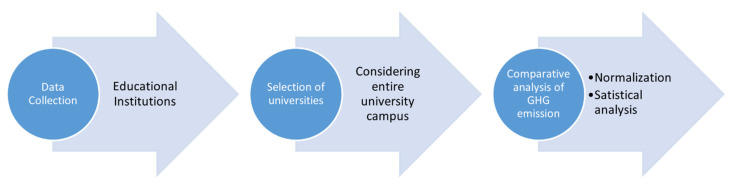
Flow chart of the research process.

The methodology used to evaluate the environmental performance in climate change mitigation of Spanish HEI through the evolution of their CF consisted of three main steps: (1) data collection, (2) filtering and selection of universities, and, finally, (3) an interpretation of the results and mapping analysis (Figure 1).

A first step to develop a standardized overall mapping was to identify all the CFs registered in the Spanish Carbon Footprint Registry (SCFR) submitted by HEIs. Emission data was obtained from the Ministry of Ecological Transition and Demographic Challenge of the Spanish Government. 

From 5544 CFs registered by 2338 organizations in the period between March 2014 (when the SCFR was created) and May 2022, the results were filtered by sector, isolating those belonging to the education sector, with 160 CFs registered (2.9% of the total). After this, educational institutions that were not universities (such as primary or secondary schools, vocational training institutes, etc.) were discarded. The sample was reduced to 81 CFs, which represents 1.5% of the total number of CFs in the SCFR, registered by 14 different Spanish HEIs (Table 1), which represents 17.1% of all Spanish HEIs [36]. This value is far behind other sectors such as transportation and warehousing (11.5%) or the manufacturing industry sector (19%), although the total number of organizations in these sectors is several thousand times higher [37].

Based on the declared activity, organizational boundaries (e.g., all campuses and their facilities, all buildings of a single campus, only one faculty or school, etc.) and the operational boundaries (e.g., teaching, research, maintenance, etc.) were established for each CF. To preserve the homogeneity of the sample, partial CFs of HEIs whose boundaries did not include the whole university (such as only one campus, or a single faculty or school) were also discarded. In addition to the emission values, the mapping of the selected data was completed by considering aspects and characteristics of each university, such as the origin of the funds (public or private) or the type of teaching (face-to-face or distance learning), location, etc. 

After collecting, reviewing, and supplementing the data, a first analysis was performed, focused on the reported information, delving into specific aspects of the calculation methodology, such as the scopes included, the seals and percentages of reduction and compensation obtained, or the reported activity rates. 

The next step consisted in a comparative analysis of the GHG emissions results obtained. To do this, it was necessary to carry out a prior normalization of the results using identical functional units or activity ratios: number of official members of the university community (disaggregated by number of students enrolled in official studies, number of teaching and research staff, and number of administrative staff) and the annual budget for each year and university.

Normalization is the calculation of the magnitude of the results of an indicator in relation to some reference information, with the aim of better understanding the relative magnitude of each result [34]. This process is also known as standardization, depending on the discipline. For a greater clarity, the term normalized is used in this article to refer to the process of transforming absolute values of GHG emissions to values relativized by some business metric that results in a ratio indicator [38]. On the other hand, the term standardization is reserved to refer to the process of making something conform to a pre-established standard or norm.

In this sense, on this study not only the results were normalized, but also the definitions of the functional units were pre-adjusted in a standardization process to ensure homogeneity and comparability of the results. For this purpose, the figures for the standardized functional units were obtained from official sources of the Spanish Government [39] and from specific activity reports published by the universities themselves [40,41,42,43,44,45,46,47,48,49,50,51,52,53,54,55].

The relationship between the CFs of the universities with respect to the different variables proposed (standardized student numbers, standardized staff numbers, total university members, and annual budgets) was analyzed using principal component analysis (PCA). These statistical analyses were performed in RStudio v.3.6.2 [56] using the RStudio base function glm and the FactoMineR package [57].

## 3. Results and Discussion

### 3.1. Registration Dates and Calculation Years

**Figure 2:**
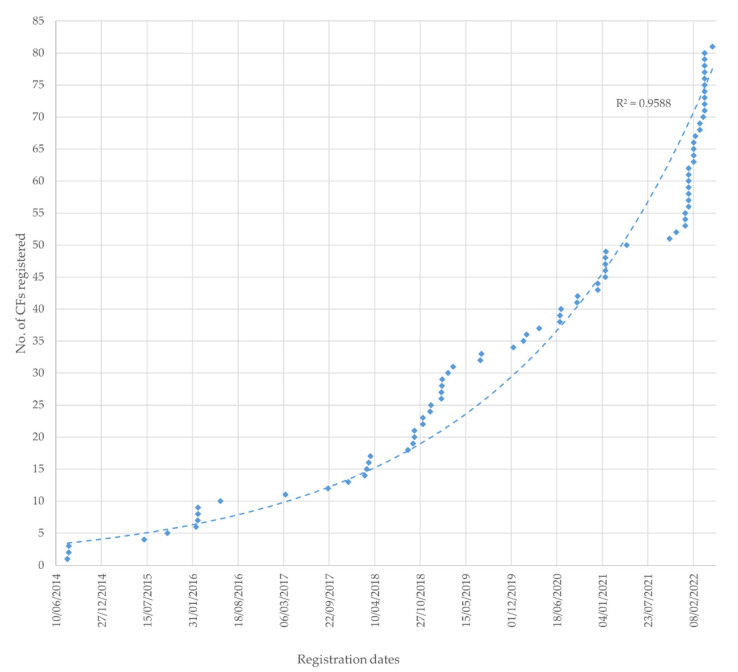
Evolution over time of the number of CF registrations by HEIs in the SCFR from its creation in March 2014 to May 2022.

From the analysis of the dates of registration of HEIs CFs, an exponential increase in the use of the Spanish Carbon Footprint Registry (SCFR) tool is observed (Figure 2). For example, 80.6% of registrations have been made in the period 2018–2022, and 35.8% of the total during the year 2022. This exponential increase in the calculation of CF and the use of SCFR is in line with the high increase that has been seen in the application of sustainable development principles in HEIs [58].

**Figure 3:**
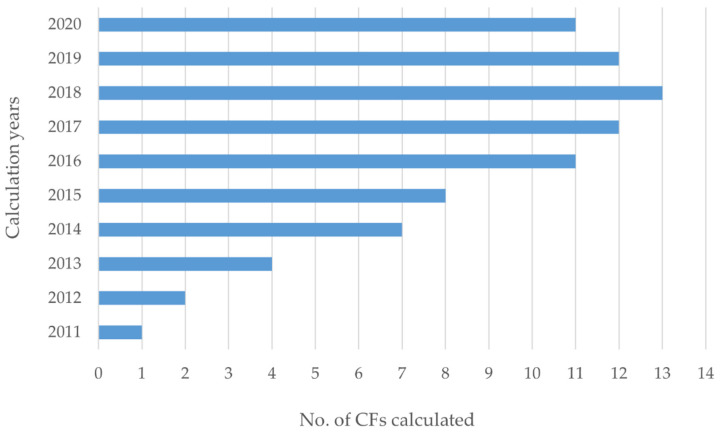
Number of CFs per calculation year.

Regarding the years calculated, although the registry was created in 2014, the CFs have been calculated for emissions from 2011 to 2020 (Figure 3). Several universities have registered at the same time their CFs for several previous years. This could be motivated by the fact that the Spanish Government requires the registration of at least 4 years of CFs to qualify for the seal that accredits a reduction of the institution’s CF.

It is noteworthy that out of the 29 CFs registered between January and May 2022, none of them calculated emissions for the previous year 2021, as would be expected. These CFs registered in 2022 included emissions for 2020 and previous years. This delay in calculation and registration is due to the fact that the emission factors for electricity trading companies for a given year are published by the Spanish Government approximately in April of the following year [59], which ensures the standardization of emission factors, but it delays the start of the calculation and registration process.

According to the results obtained by the University Sustainability Evaluation Working Group (Spanish acronym GESU), through its tool for self-diagnosis of environmental sustainability in Spanish universities, it is estimated that the total number of Spanish HEIs that calculate their CF is much higher than the 14 universities registered in the RSHC: For the year 2021, after the participation of 43 Spanish universities, it was obtained that 55.8% include global monitoring indicators such as GHG emissions, ecological footprint, CF, etc. [60]. After filtering the 43 universities participating in the GESU study against the 14 universities participating in the SCFR, it was found that there are at least 12 Spanish universities that calculate their CF but do not register it in the SCFR. It would be interesting to study the causes of this gap in depth.

Previously to this study, only a few HEI CFs were known to exist through their publication in scientific journals. The results obtained suggest that the actual number of HEIs worldwide that calculate their CFs, as part of their efforts in the fight against climate change, is much higher than that found in the scientific literature.

### 3.2. Reduction and Offsetting Seals Granted

In terms of the types of seals granted by the Spanish Government, on 27 occasions the seal obtained included at least the reduction level (25 times “Calculate and Reduce” and 2 times “Calculate, Reduce, and Offset”), demonstrating a significant reduction in CF in recent years by complying with the following formula:

$$CF_{i}+CF_{i-1}+CF_{i-2}<CF_{i-1}+CF_{i-2}+CF_{i-3}$$

where the relative *CF* for the year for which the possible reduction of *CF* is calculated (*CF_i_*), it is compared with the relative *CF* of the previous three years (*CF_i_*_−1_, *CF_i_*_−2_ and *CF_i_*_−3_).

At the level of the organization, 78.6% of the HEIs that registered their CFs achieved at some point at least one “Calculate and Reduce” seal. This significant and lasting reduction over time consolidates what has already been observed in the review of the literature on CFs in HEIs in the world [16].

This fact could demonstrate the technical effectiveness of CF assessment as a tool to detect key points for improvement [27,61,62], and its usefulness in providing a baseline against which to assess the effect of climate change mitigation actions on [5,63].

On the other hand, only two institutions (14.3%) have obtained the level 3 “Calculate, Reduce, and Offset” seal. These are apparently very low figures, but it should be taken into account that collaboration in CO_2_ absorption projects, or own tree planting, although historically suggested as a measure to offset the CF of HEIs [64,65,66], in the most recent HEI CF studies [67,68,69], it is still proposed as a hypothetical tool and not as an implemented measure. Helmers et al. [23] and Valls-Val and Bovea [16], in two literature reviews of HEIs worldwide that publish their CFs, found that only 10.0% of 20 universities and 14.3% of 35 universities, respectively, offset part of their GHG emissions, indicating that Spanish HEIs are at the same level (14.3%) as the global figure observed.

The amounts of CO_2_eq that were offset by University of Zaragoza and San Jorge University (privately managed) range from 0.04% to 4.69%, respectively. This range is lower than that observed globally by Valls-Val and Bovea [16], which ranged from 0.09% to 18%.

Regarding the way it was carried out, they compensated through collaboration with entities that implement absorption projects based on tree plantations and recognized by the Ministry. In the literature review, we found a similar example with the case of Monash University (Melbourne) which offset 18,883 t CO_2_eq by planting native forests in collaboration with an approved GHG reduction provider [70]. Other forms of offsetting are possible, such as the University of Maryland, which offset some 50,000 t CO_2_eq by purchasing carbon credits [71].

Therefore, this study could be demonstrating that universities are finally starting to implement this measure, at least in Spain, which could be mainly due to the facilities offered by the Spanish Government to “connect” the Universities with institutions that offer CO_2_ offsetting projects.

### 3.3. Organizational Boundaries

The analysis of the established organizational boundaries reveals that 75 of the 81 CFs (92.6%) included all campuses, faculties, and schools of the university. The other six CFs, that did not include the entire university, correspond to the Polytechnic University of Madrid: For the years 2011 and 2012, the CFs were calculated for only one of its technical schools, which represented less than 3% of its university population. For the years 2013 and 2014, two separate CFs were calculated per year: one for that technical school, and one for the rest of the university, being the sum of both organizational boundaries the whole university. 

**Figure 4:**
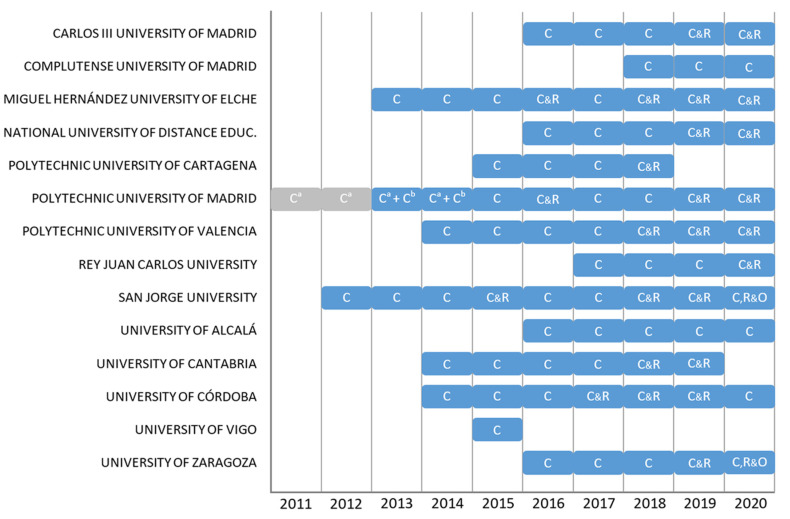
CFs registered by HEI and seals obtained (C = Calculate; C&R = Calculate and Reduce; C,R&O = Calculate, Reduce, and Offsetting); ^a^ The organizational boundary covered only one technical school of this university; ^b^ The organizational boundary covered the whole university except one technical school.

For this study, standardization of organizational boundaries from all CFs to the whole university was extremely important in order to subsequently normalize the results of relative CF and make data comparable. For this reason, those CFs whose organizational boundaries did not include the whole university were discarded, and the two CFs of the Polytechnic University of Madrid for the year 2013, as well as for 2014, were added together so that the organizational boundary was the whole university. As a result, 77 CFs from 14 Spanish HEIs with organizational boundaries that include the entire university were finally obtained for the analysis (Figure 4).

### 3.4. Operational Boundaries

With regard to operational boundaries, the 77 CFs analyzed included Scope 1 (all direct emissions) and Scope 2 (indirect emissions from electricity purchased and used by the HEI). Although the SCFR requires separate reporting of direct emissions from different types of sources (emissions from stationary installations that consume fossil fuels, emissions due to travel in own vehicles, and emissions due to the leakage of fluorinated refrigerant gases from air conditioning equipment), it has been observed that the Spanish Government does not publish this information desegregated, providing only the final value for the total of direct emissions or Scope 1. It is noted that the SCFR provides a standard for which emission sources correspond to which scope. Thanks to this standardization, the SCFR prevents universities from counting the leakage of refrigerant gases as Scope 3 [72] instead of Scope 1, or from counting the purchase of fertilizer in Scope 1 [20,73] instead of Scope 3, for example.

On the other hand, for Scope 3 (other indirect emissions), which include purchased goods and services, business travel, employee commuting, waste disposal, transport, and distribution upstream and downstream, etc. [19], its presence is almost inexistent: it was only calculated for one year by one university; moreover, the organization boundary included was only one technical school [25]. It should be noted that due to the almost infinite heterogeneity and breadth of Scope 3, quantifying it can be quite challenging for HEI, particularly for the largest and highly decentralized ones [16], and in addition, the calculations must be verified by an independent third party for registration in the SCFR. A CF study of De Montfort University in the UK [74] concluded that it is important to recognize the limitations of the CF methodology in calculating a university’s Scope 3, such as not reflecting differences between whether paper consumption is virgin or recycled, or differences in waste management practices. While emissions from energy consumption can be accurately measured, the questionnaires on which the quantification of the impact of commuting to the university is based provide relatively weak estimates due to participation bias [23]. Furthermore, in the case of transportation, the efficiency (or not) of the car drivers (without taking into account the type of fuel, engine, etc.) alone can account for a 15% variation in fuel consumption, and therefore, a 15% variation in GHG emissions with respect to the estimate [75]. Therefore, Scope 3 is far from the desired standardization of this article [76], so it was decided to focus the study of CF associated only with Scope 1 and Scope 2, discarding references to Scope 3. 

### 3.5. Characteristics of Institutions

(a) Type of learning: As shown in Table 1, 13 of the universities with registered CF are face-to-face or in-person education (although they also have some mixed or distance degrees). Only one of the universities is officially recognized as non-face-to-face or distance learning university (National University of Distance Education). The official annual average of Spanish HEIs with face-to-face education for the study period was 76 organizations, so the study sample represents 17.1% of the total number of face-to-face Spanish universities. On the other hand, the total number of universities with distance education is six, so the sample includes 16.7% of the non-face-to-face organizations. No CF data from distance education HEIs has been found in any of the literature reviews [16,20,23,77]; therefore, this mapping of the CFs of Spanish HEIs is a useful contribution to the comparative study of the contribution to climate change of distance learning universities versus face-to-face universities.
(b) University sizes: The sample ranges from small universities (with about 5000 members per year on average) to the largest of the face-to-face Spanish universities (Complutense University of Madrid, with an average of more than 80,000 members per year) as well as Spain’s largest non-attending university (the National University of Distance Education, with an average of almost 150,000 members per year). The remaining organizations range in size from 10,000 to 40,000 members per year on average. So, the different sizes of the HEI included in the study can also be considered representative.

In terms of the number of university students (official studies) included in the sample, an average of 259,197 students per year was obtained, which represents 16.6% of the average number of total students of Spanish HEIs, and this figure even rises to 28.1% for years such as 2018 (Table 2).

Considering that the number of students from distance learning universities should not be taken into account for the study, as the CF does not collect their emissions from home, if only face-to-face students are analyzed, the sample representativeness figures are still favorable, with an average of 13.5%, and a maximum in 2018 of 22.6% of the total number of Spanish HEI face-to-face students.

**Figure 5:**
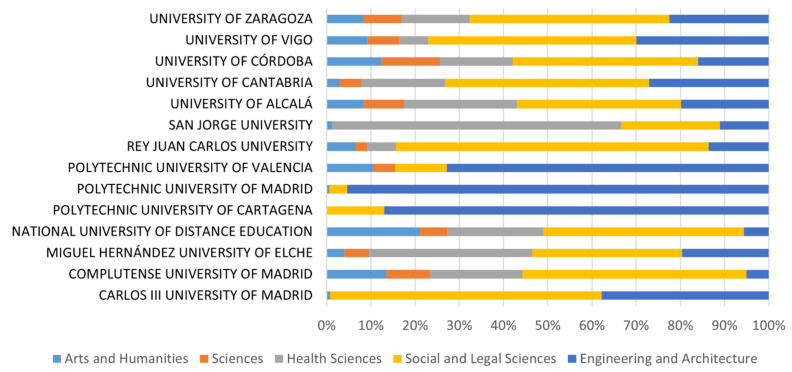
Percentages of the branches of education for each HEI in the study.

(c) Source of funding: In relation to the source of funding, one of the universities is private (San Jorge University), while the rest operate with public funds. This interferes with the fact that budgets and expenditures are not accessible, as is the case with public universities.
(d) Territorial distribution: Moreover, they are well distributed throughout the Spanish territory, with presence in 7 of its 12 autonomous communities. Although a priori this variable could be interpreted as relevant, while there is a variation in heating or air-conditioning consumption depending on weather conditions [78], recent studies have not found a correlation of CF with geographical latitude [23].
(e) Educational branches: As a result of the analysis of the different educational branches of the HEIs in the sample, it was observed that all sectors are well represented (Figure 5).

Teaching and research in science, engineering, and medicine have higher GHG emissions than humanities and social sciences [79], and office and teaching areas have lower emissions than laboratory and research spaces [80]. However, in the statistical analysis of the correlation between the different educational branches (Arts and Humanities, Sciences, Social and Legal Sciences, Health Sciences, and Engineering and Architecture) and the different results of direct GHG emissions, both relative (per student, per employee and per budget) and absolute, no correlation was found. This does not mean that, for example, science degrees consume the same amount of energy as humanities degrees, but that the choice of energy sources with lower GHG emission factors does not depend on the education branch, but on the decision making of government bodies. 

### 3.6. Total Contribution to Climate Change: Absolute CFs

The total annual GHG emissions, expressed in absolute values of CF, vary widely among HEIs CFs reported: from 213.19 t CO_2_eq (San Jorge University 2020) to 19,153.55 t CO_2_eq (Polytechnic University of Valencia 2015), with a coefficient of variation equal to 89.4%. It is therefore recommended that they be relativized for comparison.

**Figure 6:**
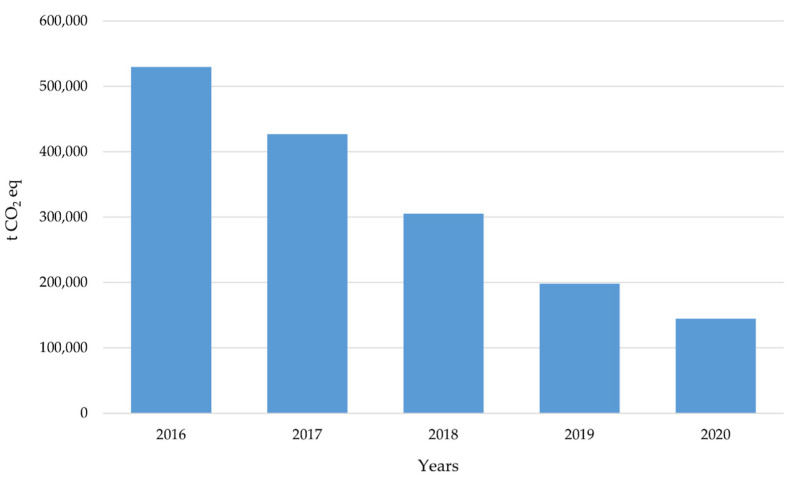
Total extrapolated greenhouse gas (GHG) emissions of the whole Spanish university system (Scopes 1 and 2).

Being aware of the likely bias that HEIs with positive GHG emission reduction policies and results are those that are more attracted to register and publish their footprints in SCFR, the study collects a sufficiently significant sample (Table 2) to allow us to extrapolate the CF results for the whole Spanish university system. Considering that for the period 2016–2020 the study sample represented between 14% and 23% of the total population in terms of university members (employees plus in-person students), the value of the total CF of the Spanish university system can be obtained (Figure 6). A clear decrease (72.7%) of the total climate change contribution of the whole Spanish university system can be observed. The subsequent detailed study of Scopes 1 and 2 separately will provide further information to explain this decline.

### 3.7. Activity Ratio Indicators

In addition to the reporting of absolute emissions, the SCFR standard requires the reporting of activity ratio indicators or functional units used to relativize the CF results. These activity ratios are freely chosen by each organization. In general, there are four main types of activity ratios chosen to calculate relative CF: students (used by six universities), persons or members (four universities), budget (one university), and degrees (one university). 

In order to find out whether these are standardized ratios or not, the figures provided were compared with figures obtained from official sources:

1. Regarding the number of students, large differences are observed in the values provided by the universities in the SRCF with respect to the figures for students in official degrees obtained from the Ministry of Universities: from −8.3% to 63.2%. This points to the fact that some universities seem to add only students from official bachelor’s and master’s programs for the calculation of the CF, while others also include postgraduate students, and others also include all students from non-official courses.
2. With regard to the number of persons or members, there is also no consensus or standardization as to what type of relationship with the university is included in this term. Although a priori it might be expected that this value would coincide with the number of members of the university community (administrative staff, teaching and research staff, and students of official degrees), on the contrary, large differences are observed in the values of persons provided by the universities with respect to the number of members: from −56.6% to 31.1%. Detailed analysis reveals that in addition to the aforementioned differences in the type of students included, there are universities that seem to include only administrative staff and teaching and research staff, while others also include workers from subcontracted companies operating on campus. San Jorge University proposes the use of the so-called “equivalent person” ratio, which could be interpreted as an attempt to equate full-time staff with part-time staff, or undergraduate students with students on courses of less than one year, among other possible equivalences [14].
3. One university chose to relativize its CFs by the number of euros in its annual budget, which undoubtedly provides a figure that is easy to monitor and compare with other universities (especially public universities). It is therefore not surprising that the values provided by the institution coincided exactly with the official values available.
4. Finally, with regard to the use of the number of degrees offered as an index of activity, this was used by the only distance learning university in the study. It is understandable that distance learning universities do not consider the number of students to be representative for monitoring their CF.

The large variations observed in terms of the interpretation of the concept of “student” (from −8.3% to 63.2%) and the concept of “university member” (from −56.6% to 31.1%) demonstrate the urgent need for consensus or standardization. Furthermore, in the literature reviews on the CF of HEIs [16,20,23,77], there is no effort to define and standardize these concepts, so the values obtained from each university of their relative CF in CO_2_eq/person or CO_2_eq/student might not be as comparable as intended.

The study of university CF by budget has been the ratio with the least variability in its definition, especially for public universities. Even so, Helmers et al. [23] proposes an adjustment in the concept, by subtracting salary payments from expenditure.

As a result, the standardization of activity ratios is proposed. These indicator definitions are not intended to be exhaustive, as more precise ratios such as “full-time equivalent person” [77], “total number of users of the facilities”, “number of chairs of each building” [22], or “overhead costs minus staff salaries” [23] would complicate the calculation. Therefore, this standardization of activity ratios is mainly based on the robustness of the data along the time and their ease of collection:

5. Standardized “number of students”: Students of official bachelor’s, master’s, and doctoral programs. Other students on non-official courses and activities are not included. In the case of universities with a high percentage of non-face-to-face students (or all of them), it is advisable not to include them.
6. Standardized “number of employees”: Administrative and service staff, teaching and research staff, and research staff. If it is decided to include workers from subcontractors operating on campus such as gardeners or maintenance staff, this should be clearly identified.
7. Standardized “number of members”: The sum of standardized “number of students” and “number of employees”.
8. “Millions of Euros annual budget”: Includes the total initial budget approved at the beginning of the year by the universities. This data has the handicap that it is not usually disseminated by private HEIs.

### 3.8. Normalized CF with Standardized Activity Ratios

Due to the lack of consensus in the definitions of activity ratios (as student or member) demonstrated in this study, the relative CF values reported by HEIs differ from the values calculated for standardized activity ratios. In the case of relative CF per student, differences ranging from −39% to 9% are observed, while for relative CF per university member, the differences range from −24% to 131%. This large uncertainty may have negatively affected the comparison of relative CFs in the existing literature reviews HEIs [16,23], where it is assumed that all universities define “student”, “employee”, or “member” in the same way, but this is not the case.

For the study, the normalized CFs provided by the Spanish HEIs were discarded, and the CFs were recalculated from standardized activity ratios obtained from official sources such as Ministries or synthesis reports from the universities themselves. (Table 3). The literature review did not find such a comprehensive list of HEIs’ CFs disaggregated by type of emission source, which is expected to be useful for future environmental sustainability assessments.

Thus, with the unified criteria for normalizing the relative results proposed in this study, this CF calculating tool, in which the calculations are verified by the Spanish government or an independent verifying entity and the results are made public, would be classified within sustainability assessment tools that allow for the generation of comparative data that can be aggregated into an overall performance rating for benchmarking purposes [81].

Finally, under the hypothesis that larger universities might have more facilities such as swimming pools or sports centers, so that their CF per person might be higher, correlations between the size of the university (in standardized number of members) and the relative CF per member were also investigated. The result obtained showed that there is no correlation in this sense (Pearson correlation coefficient equal to −0.17 and R-squared (R2) equal to 0.03), ruling out small correlations observed by some authors [23].

**Figure 7:**
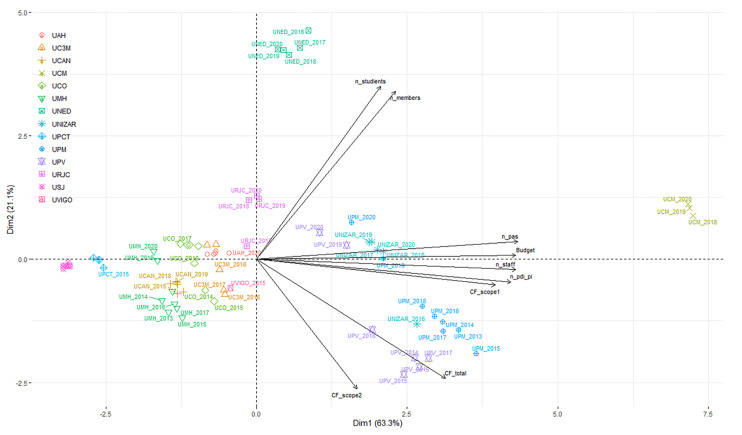
Scores and loadings for PCA performed for HEIs CFs (CF_total, total CF; CF_scope1, CF Scope 1; CF_scope2, CF Scope 2; n_students, number of students; n_pas, number of administrative and service staff; n_pdi_pi, number of teaching and research staff and research staff; n_staff, total number of employees; n_members, total number of members; budget, annual budget).

After the normalization of the data, a principal components analysis (PCA) has been made with all the activity ratios (students, administrative and service staff, teaching and research staff, and research staff, total employees, total members, and annual budget) and all the analyzed universities in this work (Figure 7). This PCA shows the distribution of the universities influence by different variables. It is clear that this model has separated the only non-presential university (UNED) from the others. Regarding the behavior of data, the figure shows that the first component (Dim1), that explain 63.3% of the variability, positively grouped with high loadings the variables n_pas, budget, n_staff, n_pdi_pi, and CF_scope1, and these variables are highly correlated with UPM, UPV, and UNIZAR. In the second component (Dim2), that explains 21.1% of the variability, the variables n_students and n_members are positively correlated and influenced by the UNED (all the years) and negatively correlated with the variables CF_total and CF_scope2. This PCA shows a high level of dispersion among the Spanish universities studied in this work, which makes it difficult to draw conclusions, but served as a basis for a second phase of statistical analysis of correlation between different parameters.

### 3.9. Correlations between Parameters

Correlations were investigated between the following parameters: total t CO_2_eq, t CO_2_eq from direct emissions (Scope 1), t CO_2_eq from electricity consumption (Scope 2), standardized number of students, standardized staff number, total university members, and annual budgets (Table 4).

**Figure 8:**
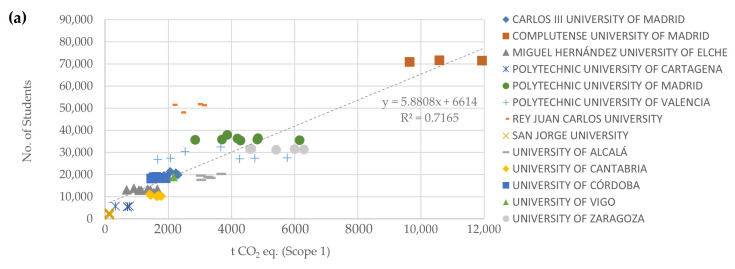

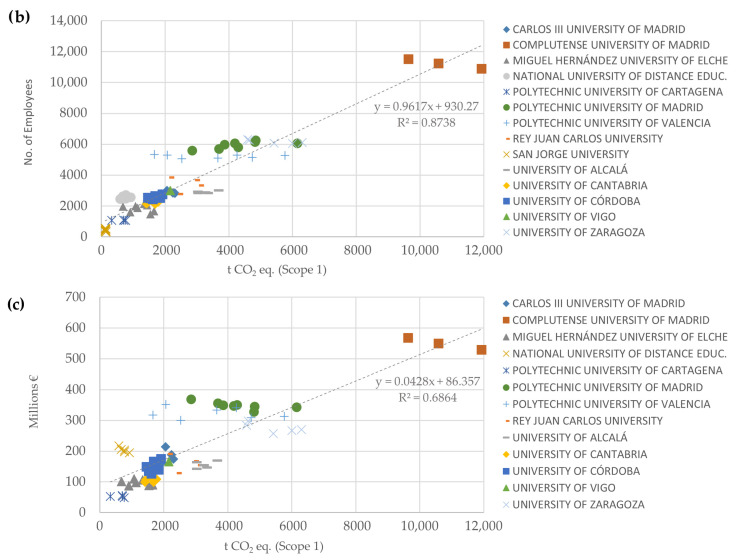
Annual total GHG direct emissions (Scope 1) reported by Spanish HEIs versus: (**a**) Standardized number of students (official bachelor’s, master’s, and PhD programs) in-person learning; (**b**) Standardized number of employees (admin. And services staff, teaching and research staff, and research staff); (**c**) Standardized annual budget of non-private universities (EUR 1 = USD 1.18 average 2013–2020 [82]).

Of 12 correlation combinations investigated, the strongest were those related to direct emissions (Scope 1) due to consumption of fossil fuels such as natural gas, diesel, etc., or leakage of refrigerant gases, both associated with the annual budget and with the standardized number of students, employees, and consequently with the total number of members (Figure 8a–c). These figures confirm that larger universities consume more fossil fuels and have a greater amount of equipment in which refrigerant gases leak occasionally. It is noteworthy from this result that in the analysis of the total carbon footprint (Scope 1 plus Scope 2) the result is not significant, so it would be advisable to evaluate and analyze carbon footprints separately broken down by scope whenever possible.

So, there was no correlation between the size of the institution, in terms of standardized number of students (official bachelor’s, master’s, and PhD programs), and the absolute values of CF reported by HEIs. As for the standardized number of employees (administration and services staff, teaching and research staff, and research staff), there was no correlation with the absolute values of HR (Scopes 1 and 2). Regarding the annual budget of the universities (known data for all non-private HEIs), there was no correlation with the absolute values of CF (Scope 1 and 2) reported.

In contrast, what could be surprising was the very weak or non-existent correlations obtained for indirect emissions due to the purchase of electricity (Scope 2) and the rest of the variables. This result confirms what was observed in case studies [28], where it was shown that GHG emissions from electricity consumption are not correlated with the amounts of electricity consumed but are strongly correlated with the source of electricity and its emission factor (Table 5). Since the emission factor varies depending on the electricity supplier, Spanish universities could reduce their CF only by contracting electricity with guaranteed renewable sources of origin.

### 3.10. Temporal Evolution

**Figure 9:**
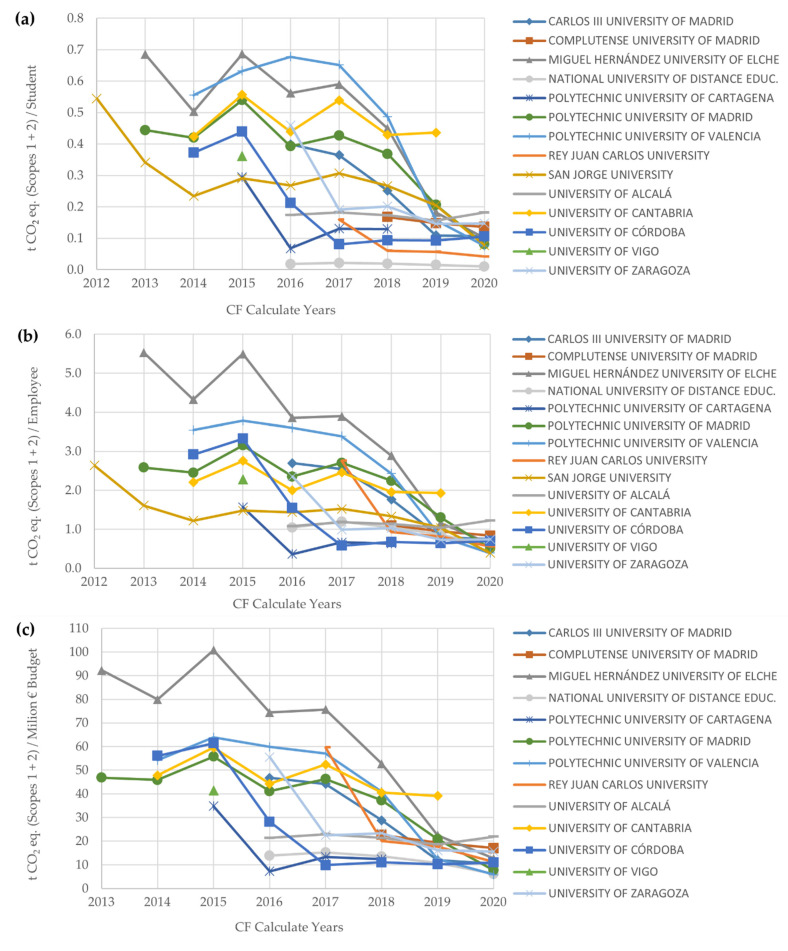
Relative CFs (Scopes 1 and 2) reported by Spanish HEIs per: (**a**) Standardized number of students (official bachelor’s, master’s, and PhD programs) in-person learning; (**b**) Standardized number of employees (admin. and services staff, teaching and research staff, and research staff); (**c**) Standardized annual budget of non-private universities (EUR 1 = USD 1.18 average 2013–2020 [82]).

With respect to the relative or normalized CFs (Scopes 1 and 2) by the standardized ratios, there is a clear tendency to decrease over the years, both for the ratios of students and staff ratios, as well as the annual budget ratio (Figure 9a–c).

**Figure 10:**
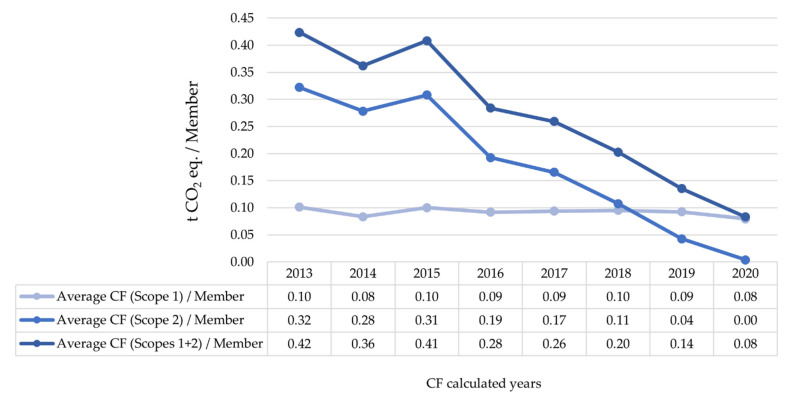
Temporal evolution of normalized CFs annual averages (by Scopes) per standardized members of Spanish HEIs registered in the SCFR.

The average relative CFs of the sample (Scopes 1 and 2) confirm the downward trend in GHG emissions by Spanish HEIs. This decrease is clearly observed in the CF per member (Figure 10), but the same results have also been obtained in the CF per student, per employee, and per budget (all of them previously standardized). In all cases, the decrease is due to a reduction in indirect GHG emissions from electricity consumption (Scope 2), while direct GHG emissions (Scope 1) remain stable.

This reduction in Scope 2 can be due to both (1) a reduction in electricity consumption and (2) an increase in the proportion of electricity consumed from renewable energy sources with renewable energy certificates [20,28,73,83,84]. The fact that this reduction is total (Scope 2 equal to zero) in many cases (32% of the CFs) suggests that the decrease in electricity consumption was not the main cause, but that all electricity comes from renewable sources with an emission factor equal to zero. 

Worldwide detailed studies on CFs in HEIs make comparisons of normalized CFs, but also include Scope 3. Indirect GHG emissions in Scope 3 are practically unlimited, both for upstream emissions (e.g., emissions from the purchase of pencils can be included depending on their raw material, the origin of the raw material, etc.) and downstream emissions (e.g., emissions from toner waste depending on the place it is taken to, the treatment it receives, the type of energy used, etc.) practically ad infinitum. Therefore, it does not seem fair nor equitable to compare the HEIs’ CFs including Scope 3, as it penalizes those universities that have produced a more complete or comprehensive report by including more Scope 3 indirect emission sources. Moreover, Scope 3 indirect emissions represent the main source of CF in most HEIs, reaching 70–80% in many cases [26,61,74], and this figure could increase if more upstream and downstream emission sources are added to the magnitude (e.g., energy consumption by students at home while studying could be added). Furthermore, these are emissions that, although they are a consequence of the university’s activity, are produced by sources that are not owned or controlled by the university, so their capacity for modification is practically nil and is reduced to recommendations in most cases.

## 4. Conclusions

- This study has made possible to draw a general map of the environmental performance in climate change mitigation of Spanish universities through a standardized tool for calculating and reporting CF. The environmental management performance in HEIs has been demonstrated by the high percentage of HEIs that calculate and record their CF compared to other economic sectors. Moreover, in recent years it has increased exponentially, on the line with the increased involvement of the world’s HEIs in the principles of sustainable development.
- The evolution of absolute values obtained for GHG emissions as an indicator of the operational environmental performance of the Spanish university system have allowed estimating a significant reduction (72.7%) for the study period, going from almost 600,000 t CO_2_eq in 2016 to about 145,000 t CO_2_eq by 2020. These data show the great effort of the Spanish universities to reduce their emissions, although there is still a significant margin for improvement in the area of offsetting GHG emissions through reforestation projects and other CO_2_ sinks.
- Absolute direct emissions (Scope 1), due to the consumption of fossil fuels such as natural gas, diesel, etc., or refrigerant gas leakage, are correlated with the annual budget and with the standardized number of students and employees. In contrast, there are no significant correlations between indirect emissions due to the purchase of electricity (Scope 2) and the other variables. Therefore, GHG emissions from electricity consumption are not correlated with the amounts of electricity consumed but are correlated with the source of electricity and its emission factor. Since the emission factor varies depending on the electricity supplier, universities could reduce their CF drastically only by contracting electricity from renewable sources. Although there is an overall decrease in total CF, direct GHG emissions (Scope 1) remain stable, so HEIs should focus their efforts on reducing the consumption of fossil fuels in their facilities, replacing them with renewable energy sources or biomass consumption.
- Regarding CF as a benchmarking tool among universities, there is a very high lack of consensus or standardization in the definition of the concepts of “student”, “employee”, and “member” used for the calculation of CF activity ratios, due to the fact that each university includes in its count, or does not include, different types of students (e.g., students in non-official studies, doctoral students, etc.) and employees (e.g., employees of outsourced companies, part-time staff, etc.). This variability causes large variations in the relative CF values per member (between −24% and 131%), depending on how the concept of “member” is defined, which is significant enough to preclude comparison between published CF values without first checking that the functional units are defined and calculated in the same way.
- Consequently, standardization of university activity ratios is proposed. These indicator definitions should be based primarily on the robustness of the data over time and their ease of collection, since more precise ratios such as “full-time equivalent person” or “actual number of users of the facilities” would complicate the calculation and prevent comparison. The annual budget of the institution is one of the functional units proposed, which is not commonly used by HEIs, but provides a well-defined activity ratio related to the size of the university and its operating capacity.
- The recalculation of all normalized CFs of HEIs with standardized activity ratios has provided detailed data for GHG emissions of HEIs broken down by type of emission source, which can serve as a basis for future benchmarking of the operational environmental performance of HEIs worldwide.
- As for the CF calculation and registration tool provided by the Spanish Government, it has been proved to be a good basis for a comparative evaluation, since it does not only standardize the sources of emissions and scopes, but also provides updated emission factors and ensures homogeneity. As an opportunity for improvement, it is proposed that the organizational and operational boundaries should always include the whole institution and its activities, or that they should be clearly specified when it is not the case.
- It can be extrapolated worldwide that the calculation of CF can be a valid key indicator for benchmarking the environmental performance in the fight against climate change of HEIs of any country, provided that all CFs must (1) have included the standardized Scope 1 and Scope 2 GHG emission sources, (2) have used the same emissions factors, and, above all, (3) have obtained the activity ratios from homogeneous standardized functional units.

## Figures and Tables

**Table 1:** Spanish higher education institutions (HEIs) with carbon footprints (CFs) registered in the Spanish Carbon Footprint Registry (SCFR).

HEI Name	HEI Spanish Acronym	Type of Learning ^a^	Founds ^b^	Date of First Register	No. of CFs Registered	Years Calculated
**CARLOS III UNIVERSITY OF MADRID**	UC3M	F	Pu	30 March 2022	5	2016–2020
**COMPLUTENSE UNIVERSITY OF MADRID**	UCM	F	Pu	4 January 2022	3	2018–2020
**MIGUEL HERNÁNDEZ UNIVERSITY OF ELCHE**	UMH	F	Pu	13 October 2015	8	2013–2020
**NATIONAL UNIVERSITY OF DISTANCE EDUCATION**	UNED	D	Pu	30 March 2022	5	2016–2020
**POLYTECHNIC UNIVERSITY OF CARTAGENA**	UPC	F	Pu	26 September 2018	4	2015–2018
**POLYTECHNIC UNIVERSITY OF MADRID**	UPM	F	Pu	30 July 2014	12 ^c^	2011–2020
**POLYTECHNIC UNIVERSITY OF VALENCIA**	UPV	F	Pu	2 June 2016	7	2014–2020
**REY JUAN CARLOS UNIVERSITY**	URJC	F	Pu	10 February 2022	4	2017–2020
**SAN JORGE UNIVERSITY**	USJ	F	Prv	23 February 2016	9	2012–2020
**UNIVERSITY OF ALCALÁ**	UAH	F	Pu	18 January 2021	5	2016–2020
**UNIVERSITY OF CANTABRIA**	UCAN	F	Pu	2 October 2018	6	2014–2019
**UNIVERSITY OF CÓRDOBA**	UCO	F	Pu	18 January 2022	7	2014–2020
**UNIVERSITY OF VIGO**	UVIGO	F	Pu	18 September 2017	1	2015
**UNIVERSITY OF ZARAGOZA**	UNIZAR	F	Pu	15 December 2017	5	2016–2020

^a^ Type of learning: F (face-to-face), D (distance learning). ^b^ Founds: Pb (public), Prv (private). ^c^ Two partial UPM CFs were recorded for two years.

**Table 2:** Percentages of students included in the CF calculations by type of HEIs.

		All HEIs			Face-to-Face HEIs			Non-Face-to-Face HEIs		
CF Year	Academic Year ^a^	Total Students ^b^	No. Students Included in CFs	% Students Included in CFs	Total Students ^b^	No. Students Included in CFs	% Students Included in CFs	Total Students ^b^	No. Students Included in CFs	% Students Included in CFs
**2012**	2011/2012	1,572,617	1525	0.1%	1,345,045	1525	0.1%	227,572	0	0.0%
**2013**	2012/2013	1,548,534	49,830	3.2%	1,327,345	49,830	3.8%	221,189	0	0.0%
**2014**	2013/2014	1,539,709	114,150	7.4%	1,311,822	114,150	8.7%	227,887	0	0.0%
**2015**	2014/2015	1,506,179	134,946	9.0%	1,279,550	134,946	10.5%	226,629	0	0.0%
**2016**	2015/2016	1,548,369	336,953	21.8%	1,315,688	180,752	13.7%	232,681	156,201	67.1%
**2017**	2016/2017	1,564,943	379,369	24.2%	1,330,150	231,971	17.4%	234,793	147,398	62.8%
**2018**	2017/2018	1,583,025	444,070	28.1%	1,345,417	304,097	22.6%	237,608	139,973	58.9%
**2019**	2018/2019	1,599,050	442,450	27.7%	1,353,605	301,666	22.3%	245,445	140,784	57.4%
**2020**	2019/2020	1,626,210	429,481	26.4%	1,369,096	292,259	21.3%	257,114	137,222	53.4%
**Average per year:**		1,565,404	259,197	16.6%	1,330,858	179,022	13.5%	234,546	80,175	34.2%

^a^ As the CF are calculated for calendar years (from January to December), but the university population data are provided for academic years (from October to September), it is considered for this study that the most representative year will be the one with the highest number of months: For example, for the CF of 2014, the data of the academic year 2013–2014 are chosen, as this academic year has 9 months of the year 2014, and only 3 months of the year 2013. ^b^ Students enrolled in official degrees (bachelor’s, master’s, and PhD) in Spanish face-to-face HEI.

**Table 3:** Normalized CFs by emission sources (Scopes 1 and 2) of Spanish HEIs recalculated from standardized activity ratios and annual averages.

Annual Average/HEI & Year	CF Scope 1 per Student ^a^	CF Scope 1 per Student ^a^	CF Scope 1 per Employee ^b^	CF Scope 2 per Employee ^b^	CF Scope 1 per Member ^c^	CF Scope 2 per Member ^c^	CF Scope 1 per M€ Budget ^d^	CF Scope 2 per M€ Budget ^d^
	(tCO_2_eq/std)	(tCO_2_eq/std)	(tCO_2_eq/emp)	(tCO_2_eq/emp)	(tCO_2_eq/per)	(tCO_2_eq/per)	(tCO_2_eq/M€)	(tCO_2_eq/M€)
**AVERAGE 2012**	0.09	0.46	0.43	2.21	0.07	0.38	11.76	61.04
**AVERAGE 2013**	0.12	0.37	0.75	2.49	0.10	0.32	14.66	47.46
**AVERAGE 2014**	0.10	0.32	0.62	2.16	0.08	0.28	11.88	40.83
**AVERAGE 2015**	0.12	0.36	0.71	2.27	0.10	0.31	13.82	43.15
**AVERAGE 2016**	0.11	0.23	0.65	1.38	0.09	0.19	12.57	26.34
**AVERAGE 2017**	0.11	0.19	0.69	1.30	0.09	0.17	13.47	24.73
**AVERAGE 2018**	0.11	0.13	0.72	0.75	0.10	0.11	13.82	13.74
**AVERAGE 2019**	0.11	0.05	0.70	0.30	0.09	0.04	12.91	5.78
**AVERAGE 2020**	0.09	0.00	0.61	0.05	0.08	0.00	10.98	0.76
**UC3M 2016**	0.10	0.30	0.69	2.00	0.09	0.26	12.01	34.75
**UC3M 2017**	0.10	0.27	0.67	1.88	0.08	0.23	11.61	32.57
**UC3M 2018**	0.12	0.14	0.81	0.95	0.10	0.12	13.23	15.49
**UC3M 2019**	0.11	0.00	0.79	0.00	0.10	0.00	11.94	0.00
**UC3M 2020**	0.10	0.01	0.68	0.06	0.09	0.01	9.59	0.87
**UCM 2018**	0.17	0.00	1.10	0.00	0.15	0.00	22.59	0.03
**UCM 2019**	0.15	0.00	0.94	0.00	0.13	0.00	19.30	0.00
**UCM 2020**	0.14	0.00	0.84	0.00	0.12	0.00	17.02	0.00
**UMH 2013**	0.13	0.56	1.04	4.49	0.11	0.50	17.32	74.92
**UMH 2014**	0.07	0.44	0.56	3.76	0.06	0.39	10.40	69.52
**UMH 2015**	0.12	0.56	0.99	4.50	0.11	0.50	18.22	82.54
**UMH 2016**	0.09	0.47	0.61	3.25	0.08	0.41	11.68	62.74
**UMH 2017**	0.05	0.54	0.35	3.55	0.05	0.47	6.75	68.91
**UMH 2018**	0.08	0.37	0.54	2.35	0.07	0.32	9.76	42.94
**UMH 2019**	0.11	0.07	0.70	0.46	0.10	0.06	13.55	8.86
**UMH 2020**	0.10	0.00	0.63	0.00	0.09	0.00	12.67	0.00
**UNED 2016**	0.00	0.01	0.29	0.76	0.00	0.01	3.81	10.09
**UNED 2017**	0.00	0.02	0.26	0.94	0.00	0.02	3.28	11.94
**UNED 2018**	0.01	0.01	0.36	0.68	0.01	0.01	4.75	8.86
**UNED 2019**	0.01	0.01	0.31	0.56	0.01	0.01	3.90	6.96
**UNED 2020**	0.00	0.01	0.24	0.30	0.00	0.01	2.73	3.43
**UPCT 2015**	0.13	0.16	0.71	0.86	0.11	0.14	15.71	19.06
**UPCT 2016**	0.06	0.01	0.31	0.05	0.05	0.01	6.20	1.10
**UPCT 2017**	0.13	0.00	0.66	0.00	0.11	0.00	13.36	0.00
**UPCT 2018**	0.13	0.00	0.63	0.00	0.11	0.00	12.38	0.00
**UPM 2013**	0.13	0.31	0.78	1.81	0.11	0.26	14.07	32.71
**UPM 2014**	0.13	0.28	0.79	1.66	0.12	0.24	14.74	31.11
**UPM 2015**	0.17	0.37	1.02	2.13	0.15	0.31	18.01	37.75
**UPM 2016**	0.12	0.28	0.69	1.66	0.10	0.24	12.12	28.94
**UPM 2017**	0.10	0.32	0.65	2.06	0.09	0.28	11.08	35.17
**UPM 2018**	0.12	0.25	0.74	1.50	0.10	0.21	12.29	24.92
**UPM 2019**	0.10	0.10	0.65	0.65	0.09	0.09	10.43	10.37
**UPM 2020**	0.08	0.00	0.51	0.00	0.07	0.00	7.76	0.00
**UPV 2014**	0.11	0.44	0.72	2.82	0.10	0.38	10.98	43.17
**UPV 2015**	0.08	0.55	0.50	3.28	0.07	0.47	8.43	55.43
**UPV 2016**	0.17	0.50	0.92	2.68	0.15	0.42	15.32	44.54
**UPV 2017**	0.21	0.44	1.09	2.29	0.18	0.37	18.40	38.64
**UPV 2018**	0.06	0.42	0.31	2.12	0.05	0.35	5.23	35.64
**UPV 2019**	0.16	0.00	0.80	0.00	0.13	0.00	12.49	0.00
**UPV 2020**	0.08	0.00	0.39	0.00	0.06	0.00	5.89	0.00
**URJC 2017**	0.05	0.11	0.87	1.88	0.05	0.10	18.83	40.74
**URJC 2018**	0.06	0.00	0.93	0.00	0.06	0.00	19.96	0.00
**URJC 2019**	0.06	0.00	0.81	0.00	0.05	0.00	17.71	0.00
**URJC 2020**	0.04	0.00	0.56	0.00	0.04	0.00	11.27	0.00
**USJ 2012**	0.09	0.46	0.43	2.21	0.07	0.38	11.76	61.04
**USJ 2013**	0.09	0.25	0.43	1.18	0.07	0.21	12.59	34.74
**USJ 2014**	0.06	0.18	0.30	0.92	0.05	0.15	7.98	24.56
**USJ 2015**	0.06	0.23	0.33	1.15	0.05	0.19	8.48	29.83
**USJ 2016**	0.06	0.21	0.32	1.12	0.05	0.18	7.77	27.64
**USJ 2017**	0.06	0.25	0.28	1.24	0.05	0.21	7.33	32.29
**USJ 2018**	0.06	0.21	0.29	1.04	0.05	0.17	7.39	26.56
**USJ 2019**	0.05	0.15	0.26	0.78	0.04	0.13	6.17	18.64
**USJ 2020**	0.04	0.03	0.22	0.17	0.04	0.03	5.44	4.08
**UAH 2016**	0.17	0.00	1.08	0.00	0.15	0.00	21.36	0.00
**UAH 2017**	0.18	0.00	1.18	0.00	0.16	0.00	22.86	0.00
**UAH 2018**	0.17	0.00	1.14	0.00	0.15	0.00	21.31	0.00
**UAH 2019**	0.16	0.00	1.04	0.00	0.14	0.00	18.60	0.00
**UAH 2020**	0.18	0.00	1.22	0.00	0.16	0.00	21.85	0.00
**UCAN 2014**	0.13	0.30	0.66	1.55	0.11	0.25	14.28	33.49
**UCAN 2015**	0.16	0.40	0.79	1.96	0.13	0.33	17.10	42.43
**UCAN 2016**	0.13	0.30	0.61	1.38	0.11	0.25	13.59	30.66
**UCAN 2017**	0.16	0.38	0.74	1.71	0.13	0.31	15.84	36.55
**UCAN 2018**	0.17	0.26	0.79	1.17	0.14	0.21	16.35	24.11
**UCAN 2019**	0.16	0.27	0.71	1.21	0.13	0.22	14.48	24.58
**UCO 2014**	0.09	0.29	0.67	2.24	0.08	0.25	12.92	43.12
**UCO 2015**	0.08	0.36	0.62	2.70	0.07	0.32	11.52	49.89
**UCO 2016**	0.10	0.11	0.73	0.82	0.09	0.10	13.32	14.83
**UCO 2017**	0.08	0.00	0.58	0.00	0.07	0.00	9.85	0.00
**UCO 2018**	0.09	0.00	0.67	0.00	0.08	0.00	11.08	0.00
**UCO 2019**	0.09	0.00	0.64	0.00	0.08	0.00	10.24	0.00
**UCO 2020**	0.11	0.00	0.70	0.00	0.09	0.00	11.00	0.00
**UVIGO 2015**	0.11	0.25	0.72	1.56	0.10	0.21	13.09	28.26
**UNIZAR 2016**	0.17	0.28	0.89	1.46	0.15	0.24	21.12	34.43
**UNIZAR 2017**	0.19	0.00	0.99	0.00	0.16	0.00	22.47	0.00
**UNIZAR 2018**	0.20	0.00	1.03	0.00	0.17	0.00	23.30	0.00
**UNIZAR 2019**	0.15	0.00	0.73	0.00	0.12	0.00	16.14	0.00
**UNIZAR 2020**	0.15	0.00	0.74	0.00	0.12	0.00	15.57	0.00

^a^ Includes students of official bachelor’s, master’s, and PhD programs. ^b^ Includes administration and services staff, teaching and research staff, and research staff. ^c^ Includes students of official bachelor’s, master’s, and PhD programs plus administration and services staff, teaching and research staff, and research staff. ^d^ Annual official budget in millions of euros of public universities. The budget of the private university was estimated from the average budget per student of the rest of HEIs.

**Table 4:** Pearson correlations and R-squared (R^2^) between standardized number of students (official bachelor’s, master’,s and PhD programs), standardized number of employees (administration and services staff, teaching and research staff, and research staff), standardized number of university members (students plus employees), and annual budgets versus absolute CF values reported by Spanish HEIs.

		Absolute CF (Scope 1 + 2)	Absolute CF (Scope 1)	Absolute CF (Scope 2)
**No. Students**	Pearson correlation	0.50	0.85	0.16
	N	72	72	72
	R-squared (R^2^)	0.25	0.72	0.02
**No. Employees**	Pearson correlation	0.62	0.93	0.24
	N	77	77	77
	R-squared (R^2^)	0.38	0.87	0.06
**No. Members**	Pearson correlation	0.52	0.87	0.17
	N	72	72	72
	R-squared (R^2^)	0.96	0.76	0.06
**Millions € Budget**	Pearson correlation	0.55	0.83	0.22
	N	68	68	68
	R-squared (R^2^)	0.31	0.69	0.05

**Table 5:** Pearson Correlations between electricity consumption, emission factors, and HEI CF [28].

		Absolute CF (Scope 1 + 2)	Electricity Consumption	Emission Factor
**Electricity consumption**	Pearson correlation	0.50	1	−0.062
	Sig. (bilateral)	72		0.773
	N	0.25	24	24
**Emission Factor**	Pearson correlation	0.62	−0.062	1
	Sig. (bilateral)	77	0.773	
	N	0.38	24	24
**Carbon Footprint**	Pearson correlation	0.52	0.340	0.916 **
	Sig. (bilateral)	72	0.104	0.000
	N	0.96	24	24

** The correlation is significant at the 0.01 level (bilateral).

## Data Availability

Not applicable.

## References

[1] Gu Y., Wang H., Xu J., Wang Y., Wang X., Robinson Z.P., Li F., Wu J., Tan J., Zhi X. (2019). Quantification of Interlinked Environmental Footprints on a Sustainable University Campus: A Nexus Analysis Perspective. Appl. Energy.

[2] Cortese A. (2003). The Critical Role of Higher Education in Creating a Sustainable Future. Plan. High. Educ..

[3] Gomera A., de Toro A., Aguilar J.E., Guijarro C., Antúnez M., Vaquero-Abellán M. (2021). Combining Management, Education and Participation for the Transformation of Universities towards Sustainability: The Trébol Programme. Sustainability.

[4] Cordero E.C., Centeno D., Todd A.M. (2020). The Role of Climate Change Education on Individual Lifetime Carbon Emissions. PLoS ONE.

[5] Robinson O.J., Tewkesbury A., Kemp S., Williams I.D. (2018). Towards a Universal Carbon Footprint Standard: A Case Study of Carbon Management at Universities. J. Clean. Prod..

[6] Gómez F.U., Sáez-Navarrete C., Lioi S.R., Marzuca V.I. (2015). Adaptable Model for Assessing Sustainability in Higher Education. J. Clean. Prod..

[7] Alba D. (2015). The Evaluation of the University’s Contribution to Environmental Sustainability: An Application to Spanish Universities, Doctorate Program Interuniversity in Environmental Education.

[8] Johnstone L. (2020). The Construction of Environmental Performance in ISO 14001-Certified SMEs. J. Clean. Prod..

[9] Trumpp C., Endrikat J., Zopf C., Guenther E. (2015). Definition, Conceptualization, and Measurement of Corporate Environmental Performance: A Critical Examination of a Multidimensional Construct. J. Bus. Ethics.

[10] (1999). Environmental Management—Environmental Performance Evaluation—Guidelines.

[11] Xie S., Hayase K. (2007). Corporate Environmental Performance Evaluation: A Measurement Model and a New Concept. Bus. Strateg. Environ..

[12] Boiral O., Guillaumie L., Heras-Saizarbitoria I., Tayo Tene C.V. (2018). Adoption and Outcomes of ISO 14001: A Systematic Review. Int. J. Manag. Rev..

[13] Spencer S.Y., Adams C., Yapa P.W.S. (2013). The Mediating Effects of the Adoption of an Environmental Information System on Top Management’s Commitment and Environmental Performance. Sustain. Account. Manag. Policy J..

[14] Lo-Iacono-Ferreira V.G., Torregrosa-López J.I., Capuz-Rizo S. The Use of Carbon Footprint as a Key Performance Indicator in Higher Education Institutions. Proceedings of the 22nd International Congress on Project Management and Engineering.

[15] Ridhosari B., Rahman A. (2020). Carbon Footprint Assessment at Universitas Pertamina from the Scope of Electricity, Transportation, and Waste Generation: Toward a Green Campus and Promotion of Environmental Sustainability. J. Clean. Prod..

[16] Valls-Val K., Bovea M.D. (2021). Carbon Footprint in Higher Education Institutions: A Literature Review and Prospects for Future Research. Clean Technol. Environ. Policy.

[17] Kulkarni S.D. (2019). A Bottom up Approach to Evaluate the Carbon Footprints of a Higher Educational Institute in India for Sustainable Existence. J. Clean. Prod..

[18] Wright L.A., Kemp S., Williams I. (2011). “Carbon Footprinting”: Towards a Universally Accepted Definition. Carbon Manag..

[19] WRI/WBCSD Greenhouse Gas Protocol (2011). Corporate Value Chain (Scope 3) Accounting and Reporting.

[20] Bailey G., LaPoint T. (2016). Comparing Greenhouse Gas Emissions across Texas Universities. Sustainability.

[21] Robinson O.J., Kemp S., Williams I. (2015). Carbon Management at Universities: A Reality Check. J. Clean. Prod..

[22] Abdelalim A., O’Brien W., Shi Z. (2015). Visualization of Energy and Water Consumption and GHG Emissions: A Case Study of a Canadian University Campus. Energy Build..

[23] Helmers E., Chang C.C., Dauwels J. (2021). Carbon Footprinting of Universities Worldwide: Part I—Objective Comparison by Standardized Metrics. Environ. Sci. Eur..

[24] Valls-Val K., Bovea M.D. (2022). Carbon Footprint Assessment Tool for Universities: CO2UNV. Sustain. Prod. Consum..

[25] Alvarez S., Blanquer M., Rubio A. (2014). Carbon Footprint Using the Compound Method Based on Financial Accounts. The Case of the School of Forestry Engineering, Technical University of Madrid. J. Clean. Prod..

[26] Gomez N., Cadarso M.Á., Monsalve F., Gómez N., Cadarso M.Á., Monsalve F., Gomez N., Cadarso M.Á., Monsalve F. (2016). Carbon Footprint of a University in a Multiregional Model: The Case of the University of Castilla-La Mancha. J. Clean. Prod..

[27] Rodríguez Andara A., Rio Belver R.M., Garcia Marina V. (2020). Sustainable University Institutions: Determination of Gases Greenhouse Efect in a University Center and Strategies to Decrease Them. DYNA.

[28] Guerrero A.J., García-Orenes F., Ruiz J.J., Vicente P.G. (2019). The Spanish Carbon Footprint Calculation and Registration System: The Miguel Hernández of Elche University Case. Adv. Ecol. Environ. Res..

[29] Puchades M., De la Guardia A., Albertos J. (2011). La Huella de Carbono de La Universitat de València: Diagnóstico, Análisis y Evaluación. Cuad. Geogr..

[30] Arroyo Hernández P., Álvarez J.M., Falagán Fernández J., Martínez Sanz C., Ansola González G., Calabuig E.D.L. (2009). Ecological Footprint of the Vegazana Campus. An Approach to Its Value. Implications for the Sustainability of the University Community. Seguridad y Medio Ambiente.

[31] Torregrosa-López J.I., Lo-Iacono-Ferrreira V.G., Lledó-Lagardera D., Martí-Barranco C. Un Indicador Ambiental Para Medir La Sostenibilidad En Las Universidades, La Huella Ecológica. Caso de Estudio de La Universitat Politècnica de València. Proceedings of the National Environmental Congress CONAMA10.

[32] Giménez A., Pérez I., Montesinos P., Vera V., Bordonado S. (2009). Pautas de Movilidad y Alternativas de Reducción de La Huella Ecológica En Centros de Trabajo: La Universidad Miguel Hernández Como Caso de Estudio. Fundación MAPFRE. Seguridad y Medioambiente.

[33] Lo-Iacono-Ferreira V.G., Capuz-Rizo S.F., Torregrosa-López J.I. (2018). Key Performance Indicators to Optimize the Environmental Performance of Higher Education Institutions with Environmental Management System—A Case Study of Universitat Politècnica de València. J. Clean. Prod..

[34] (2006). Environmental Management—Life Cycle Assessment—Requirements and Guidelines.

[35] (2014). Spanish Government Royal Decree 163/2014, of 14 March, Creating the Registry of Carbon Footprint, Offsetting and Carbon Dioxide Absorption Projects.

[36] Ministry of General Secretariat of Universities Integrated University Information System. General Secretariat of Universities. https://www.universidades.gob.es/portal/site/universidades/menuitem.78fe777017742d34e0acc310026041a0/?vgnextoid=b93dd58bc3350710VgnVCM1000002006140aRCRD.

[37] INE National Statistics Institute—Companies Registered in Spain by Main Activity (CNAE 2009 Groups). https://www.ine.es/jaxiT3/Tabla.htm?t=298.

[38] WRI/WBCSD Greenhouses Gases Procotol (2004). A Corporate Accounting and Reporting Standard.

[39] Ministry of General Secretariat of Universities Data and Figures of the Spanish University System (2011–2021). https://www.universidades.gob.es/portal/site/universidades/menuitem.a9621cf716a24d251662c810026041a0/?vgnextoid=044e91d248552710VgnVCM1000001d04140aRCRD.

[40] UC3M Carlos III University of Madrid 2020 Budget. https://www.uc3m.es/about-uc3m/financial-information.

[41] UCM Complutense University of Madrid 2019–2020 Facts and Figures. https://www.ucm.es/portaldetransparencia/ucm-en-cifras.

[42] USJ San Jorge University 2013–2015 Reports. https://cultura.usj.es/ediciones/memorias-academicas/.

[43] UAH University of Alcalá 2020 Budget. https://transparencia.uah.es/transparencia-economica/presupuestos-2020/.

[44] UNICAN University of Cantabria Reports 2013–2015. https://web.unican.es/unidades/serviciodecomunicacion/publicaciones-institucionales.

[45] UCO University of Córdoba Annual Financial Reports—2014–2020. https://www.uco.es/gestion/gestioneconomica/presupuestos.

[46] UVIGO University of Vigo Social Responsibility Report 2015. https://secretaria.uvigo.gal/uv/web/transparencia/pagina/show/47?_locale=es.

[47] UNIZAR University of Zaragoza 2020 Budget. https://www.unizar.es/institucion/presupuesto.

[48] UMH Miguel Hernández University of Elche Financial Year 2020 Budget Report. https://presupuestoypatrimonio.umh.es/2019/12/19/presupuesto-2020/.

[49] UMH Miguel Hernández University of Elche Social Responsibility Reports 2007–2015. https://vdo.institucionales.umh.es/rsu/.

[50] UNED National University of Distance Education 2020 Budget. https://www.uned.es/universidad/inicio/portaltransparencia/contenidos/presupuestos-ejecucion.html.

[51] UPM Polytechnic University of Madrid Social Council Report 2013–2015. https://www.upm.es/UPM/ConsejoSocial/Documentacion.

[52] UPM Polytechnic University of Madrid—Budgets 2020. https://transparencia.upm.es/economico/memoria.

[53] UPV Polytechnic University of Valencia—Reports of the Academic Years 2013/14 and 2014/15. https://www.upv.es/entidades/SG/infoweb/sg/info/518515normalc.html.

[54] UPV Polytechnic University of Valencia—2020 Budget. http://www.upv.es/entidades/GER/info/1094557normalc.html.

[55] Rey Juan Carlos University Rey Juan Carlos University General Budget 2020. https://transparencia.urjc.es/informacion-economica/.

[56] RStudio Team (2022). RStudio v.3.6.2.

[57] Lê S., Josse J., Husson F. (2008). FactoMineR: An R Package for Multivariate Analysis. J. Stat. Softw..

[58] Caeiro S., Hamón L.A.S., Martins R., Aldaz C.E.B. (2020). Sustainability Assessment and Benchmarking in Higher Education Institutions-a Critical Reflection. Sustainability.

[59] MITECO Emission Factors (2022). Registry of Carbon Footprint, Offsetting and Carbon Dioxide Removal.

[60] (2022). GESU Crue-Sosteniblidad Environmental Sustainability Diagnosis in Spanish Universities 2021.

[61] Vásquez L., Iriarte A., Almeida M., Villalobos P. (2015). Evaluation of Greenhouse Gas Emissions and Proposals for Their Reduction at a University Campus in Chile. J. Clean. Prod..

[62] Laingoen O., Kongkratoke S., Dokmaingam P. (2016). Energy Consumption and Greenhouse Gas Emission Evaluation Scenarios of Mea Fah Luang University. MATEC Web Conf..

[63] Letete T.C.M., Mungwe N.W., Guma M., Marquard A. (2011). Carbon Footprint of the University of Cape Town. J. Energy S. Afr..

[64] Ologun O.O., Wara S.T. (2014). Carbon Footprint Evaluation and Reduction as a Climate Change Mitigation Tool—Case Study of Federal University of Agriculture Abeokuta, Ogun State, Nigeria. Int. J. Renew. Energy Res..

[65] Riedy C., Daly J. (2010). Targeting a Low-Carbon University: A Greenhouse Gas Reduction Target for the Australian Technology Network of Universities. Universities and Climate Change—Introducing Climate Change at University Programmes.

[66] Almufadi F., Irfan M.A. (2016). Initial Estimate of the Carbon Footprint of Qassim University, Saudi Arabia. Int. J. Appl. Eng. Res..

[67] Yañez P., Sinha A., Vásquez M. (2019). Carbon Footprint Estimation in a University Campus: Evaluation and Insights. Sustainability.

[68] Syafrudin S., Zaman B., Budihardjo M.A., Yumaroh S., Gita D.I., Lantip D.S. (2020). Carbon Footprint of Academic Activities: A Case Study in Diponegoro University. IOP Conf. Ser. Earth Environ. Sci..

[69] Tae H., Ko C.-S., Kwak J.-G., Seong A., Alothman M.A., Alrowaili Z.A., Iskandar J., Rahma N., Rosnarti D., Purnomo A.B. (2020). The Carbon Footprint of Trisakti University’s Campus in Jakarta, Indonesia. IOP Conf. Ser. Earth Environ. Sci..

[70] Monash University Annual Report 2016. https://www.monash.edu/__data/assets/pdf_file/0011/844508/monash-university-2016-annual-report.pdf.

[71] UMD University of Maryland Climate Action Plan 2.0uthor|Positive Change Purchasing Cooperative. https://positivechangepc.com/uncategorized/university-of-maryland-climate-action-plan-2-0uthor/.

[72] Sangwan K.S., Bhakar V., Arora V., Solanki P. (2018). Measuring Carbon Footprint of an Indian University Using Life Cycle Assessment. Procedia CIRP.

[73] Clabeaux R., Carbajales-Dale M., Ladner D., Walker T. (2020). Assessing the Carbon Footprint of a University Campus Using a Life Cycle Assessment Approach. J. Clean. Prod..

[74] Ozawa-Meida L., Brockway P., Letten K., Davies J., Fleming P. (2013). Measuring Carbon Performance in a UK University through a Consumption-Based Carbon Footprint: De Montfort University Case Study. J. Clean. Prod..

[75] Larrazábal J. (2004). La Conducción Eficiente. Dyna.

[76] Patchell J. (2018). Can the Implications of the GHG Protocol’s Scope 3 Standard Be Realized?. J. Clean. Prod..

[77] Sinha P., Schew W.A., Sawant A., Kolwaite K.J., Strode S.A. (2012). Greenhouse Gas Emissions from U.S. Institutions of Higher Education. J. Air Waste Manag. Assoc..

[78] Schwartzkopf L., Urban G. Minnesota State University (Mankato) Carbon Footprint Update Report 2018. https://mankato.mnsu.edu/globalassets/finance-and-administration/greencampus/carbonfootprint/minnesota-state-mankato-carbon-footprint-update-report-2018-final.pdf.

[79] Larsen H.N., Pettersen J., Solli C., Hertwich E.G. (2013). Investigating the Carbon Footprint of a University—The Case of NTNU. J. Clean. Prod..

[80] Klein-Banai C., Theis T.L. (2013). Quantitative Analysis of Factors Affecting Greenhouse Gas Emissions at Institutions of Higher Education. J. Clean. Prod..

[81] Fischer D., Jenssen S., Tappeser V. (2015). Getting an Empirical Hold of the Sustainable University: A Comparative Analysis of Evaluation Frameworks across 12 Contemporary Sustainability Assessment Tools. Assess. Eval. High. Educ..

[82] European Central Bank Euro Foreign Exchange Reference Rates. https://www.ecb.europa.eu/stats/policy_and_exchange_rates/euro_reference_exchange_rates/html/eurofxref-graph-usd.en.html.

[83] Baboulet O., Lenzen M. (2010). Evaluating the Environmental Performance of a University. J. Clean. Prod..

[84] Criollo N.P., Ramirez A.D., Salas D.A., Andrade R. The Role of Higher Education Institutions Regarding Climate Change: The Case of Escuela Superior Politécnica Del Litoral and Its Carbon Footprint in Ecuador. Proceedings of the ASME International Mechanical Engineering Congress and Exposition, American Society of Mechanical Engineers.

